# Mild hypothermia reduces cardiac post-ischemic reactive hyperemia

**DOI:** 10.1186/1471-2261-7-5

**Published:** 2007-02-26

**Authors:** Goran K Olivecrona, Matthias Götberg, Jan Harnek, Jesper Van der Pals, David Erlinge

**Affiliations:** 1 Department of Cardiology, Lund University, Lund, Sweden; 2 Department of Radiology, Lund University, Lund, Sweden

## Abstract

**Background:**

In experimentally induced myocardial infarction, mild hypothermia (33–35°C) is beneficial if applied prior to ischemia or reperfusion. Hypothermia, when applied after reperfusion seems to confer little or no benefit. The mechanism by which hypothermia exerts its cell-protective effect during cardiac ischemia remains unclear. It has been hypothesized that hypothermia reduces the reperfusion damage; the additional damage incurred upon the myocardium during reperfusion. Reperfusion results in a massive increase in blood flow, reactive hyperemia, which may contribute to reperfusion damage. We postulated that hypothermia could attenuate the post-ischemic reactive hyperemia.

**Methods:**

Sixteen 25–30 kg pigs, in a closed chest model, were anesthetized and temperature was established in all pigs at 37°C using an intravascular cooling catheter. The 16 pigs were then randomized to hypothermia (34°C) or control (37°C). The left main coronary artery was then catheterized with a PCI guiding catheter. A Doppler flow wire was placed in the mid part of the LAD and a PCI balloon was then positioned proximal to the Doppler wire but distal to the first diagonal branch. The LAD was then occluded for ten minutes in all pigs. Coronary blood flow was measured before, during and after ischemia/reperfusion.

**Results:**

The peak flow seen during post-ischemic reactive hyperemia (during the first minutes of reperfusion) was significantly reduced by 43 % (p < 0.01) in hypothermic pigs compared to controls.

**Conclusion:**

Mild hypothermia significantly reduces post-ischemic hyperemia in a closed chest pig model. The reduction of reactive hyperemia during reperfusion may have an impact on cardiac reperfusion injury.

## Background

Coronary reperfusion injury is believed to occur during reperfusion of an occluded coronary artery in the setting of myocardial infarction. The existence of this injury is still a matter of debate [1], but also the focus of a great deal of research on how to limit the possible additional cell death thought to occur during reperfusion [2]. The mechanism of this proposed reperfusion injury is unclear but several hypotheses have been proposed: oxygen free radical formation, calcium overload, neutrophil-mediated myocardial and endothelial injury, progressive decline in microvascular flow to the reperfused myocardium, and depletion of high energy phosphate stores [3]. Although a number of pharmacological treatments, aimed at the above mentioned mechanism, have been tried in experimental settings, none have yet yielded positive results in a large randomized trial in man.

During a coronary artery occlusion, the area of the heart supplied by the artery is deprived of its blood-supply. Upon reperfusion there is a dramatic rise in coronary blood flow, far above the baseline flow prior to the occlusion [4]. This phenomenon, post ischemic reactive hyperemia, is somewhat of an enigma because the incurred oxygen debt does not in itself justify the flow increase [5]. In experiments by Badeer et al., the excess coronary flow measured during post ischemic reactive hyperemia was 4 times that of the incurred debt [6]. Although the peak flow is greatly increased rapidly following reperfusion, it also rapidly returns to baseline within a few minutes [7]. Several factors have been implicated and the mechanism is now thought to be multi-factorial involving adrenalin, ADP/ATP, adenosine, substance P, and bradykinin but there still exists an unaccountable rise in blood flow during reperfusion [5,7-16].

Therapeutic mild hypothermia has experimentally in large animals been shown to limit myocardial infarct size if applied prior to reperfusion [17-19], but not following reperfusion [19]. It has also been shown that the earlier hypothermia is applied to an ischemic myocardium, the more tissue stands to be salvaged [20]. Post hoc analyses of the 2 large randomized hypothermia trials in man, COOL-MI (O'Neill WW, on behalf of the COOL-MI investigators. Cooling as an adjunct to primary PCI for myocardial infarction. Presented at Transcatheter Cardiovascular Therapeutics (TCT) 2003, Washington DC, USA, September, 2003) and ICE-IT (Grines CL, on behalf of the ICE-IT investigators. Intravascular cooling adjunctive to percutaneous coronary intervention for acute myocardial infarction. Presented at Transcatheter Cardiovascular Therapeutics (TCT) 2004, Washington DC, USA, September, 2004), indicates that attaining a core temperature of < 35°C before reperfusion is essential in order to reduce myocardial infarct size. Unfortunately the mechanisms by which mild hypothermia exerts its effect is still unknown, although it is a common belief that the decrease in metabolic demand in the hypothermic myocardium is one explanation for the reduced infarction size. Thus, there is a need to better understand the protective mechanisms of hypothermia, in order to design future human trials in a better way.

Since reactive hyperemia is an occurrence which immediately follows reperfusion, and thus potentially could be involved in the development of a reperfusion injury, we wanted to examine if reactive hyperemia could be affected by hypothermia. We hypothesized that the large blood flow seen during coronary post ischemic reactive hyperemia could be attenuated by mild systemic hypothermia and that this may be a mechanism by which hypothermia could reduce reperfusion injury.

## Methods

### Animals

16 healthy domestic male and female pigs weighing 25 kg were fasted overnight with free access to water and were premedicated with azaperone (Stresnil Vet., Leo; Helsingborg, Sweden), 2 mg/kg intramuscularly 30 min before the procedure. After induction of anesthesia with thiopental 5–25 mg/kg (Pentothal, Abbott, Stockholm, Sweden), the animals were orally intubated with cuffed endotracheal tubes. A slow infusion of 1.25 μl/ml Fentanyl (Fentanyl, Pharmalink AB, Stockholm, Sweden) in Ringer's acetate solution was started at a rate of 1.5 ml/min and adjusted as needed. Mechanical ventilation was then established with a Siemens-Elema 300B ventilator in the volume-controlled mode. Initial settings were: respiratory rate of 15/min, tidal volume of 10 ml/kg, and positive end-expiratory pressure of 5 cm H_2_O. Min volume was subsequently adjusted in order to obtain normocapnia (35–40 mm Hg). The animals were ventilated with a mixture of dinitrous oxide (70%) and oxygen (30%). Anesthesia was complemented with small intermittent doses of 5 mg meprobamat (Mebumal, DAK, Copenhagen, Denmark) and thiopental (Pentothal, Abbott, Stockholm, Sweden), if needed.

A 6 F introducer sheath (Boston Scientific Scimed, Maple Grove, MN, USA) was inserted into the surgically exposed left femoral artery with the tip of the introducer located in the Iliac Artery or the distal Abdominal Aorta. The side port of the introducer was connected to a pressure transducer and balanced to atmospheric pressure with zero reference at the mid-axillary level for continuously monitoring of the arterial pressure. A three-lead ECG was displayed on the same monitor as the pressure curve (78342 A, Hewlett and Packard GMBH. Boeblingen, Germany).

A 12 F introducer sheath (Boston Scientific Scimed, Maple Grove, MN, USA) was inserted into the surgically exposed left Femoral Vein. A 10.7 F cooling (temperature control) catheter (Celsius Control™, Innercool Therapeutic, San Diego, CA, USA) was inserted through the sheath and positioned in the Inferior Vena Cava.

A 12 F introducer sheath (Boston Scientific Scimed, Maple Grove, MN, USA) was inserted into the surgically exposed left External Jugular Vein. A short 10 F special catheter of our own design was used to catheterize the Azygos Vein. Then a 6 F MPA coronary catheter (Boston Scientific Scimed, Maple Grove, MN, USA) was passed through the catheter with the tip in the Azygos Vein, into the Coronary Sinus, often with the help of a PT choice guide wire, (Boston Scientific Scimed, Maple Grove, MN, USA).

A 6 F introducer sheath (Boston Scientific Scimed, Maple Grove, MN, USA) was inserted into the surgically exposed left carotid artery upon which a 6F JL 3.5 Wiseguide™ (Boston Scientific Scimed, Maple Grove, MN, USA) was inserted into the left main coronary artery and 10,000 IU of Heparin was administered. An angiogram was obtained using 8–10 ml of the contrast medium Omnipaque™ 300 mg I^-^/ml (Nycomed, Oslo, Norway) to ensure correct positioning of the catheter. The catheter was used to place a 0.014-inch, 12 MHz pulsed Doppler flow velocity transducer (Jometrics Flowire, Jomed NV) into the mid-portion of the left anterior descending artery (LAD) and a 0.014-inch PT choice™ guidewire (Boston Scientific Scimed, Maple Grove, MN, USA) into the distal portion of the LAD. A 3.0 × 20 mm over the wire Maverick™ angioplasty balloon (Boston Scientific Scimed, Maple Grove, MN, USA) was then positioned in the mid portion of the LAD, proximal to the flow velocity transducer but distal to the first diagonal branch, followed by the withdrawal of the PT choice guidewire. Continuous coronary velocity flow profiles were displayed and recorded using the Doppler flow wire connected to a FloMap monitor (Cardiometrics, Mountain View, CA). Flow was measured in units of average peak velocity (APV) in centimeters per second. All radiological procedures were performed in an experimental catheterization laboratory, (Shimadzu Corp., Kyoto, Japan). The diameter of the LAD was measured in separate pigs during baseline and during reperfusion at the same angle to ensure that a change in diameter did not occur in the LAD.

In all 16 pigs baseline registrations were performed. Regardless of initial temperature, all pigs were cooled or warmed (as needed) to a baseline temperature of 37°C, which was then maintained for 30 minutes. The pigs were then randomized to the hypothermia group or to the control group using a simple randomization by drawing folded notes out of a box which read "cool" or "warm". The pigs randomized to hypothermia were then cooled with the cooling catheter to a temperature of 34.0°C, prior to balloon inflation, which was then maintained until sacrifice. The pigs randomized to the control group were actively maintained at 37°C using the endovascular cooling catheter until sacrifice.

In all pigs, the LAD was then occluded distal to the first diagonal branch by inflation of the angioplasty balloon for a period of ten min. The coronary blood flow in the LAD was measured before, during and after occlusion of the LAD. During the reperfusion phase, flow was measured at every 10 sec. Flow was measured in average peak velocity (APV) in cm/sec. Occlusion of the LAD was performed only once in each pig.

Blood gases were collected through the MPA catheter in the Coronary Sinus and the Femoral Artery sheath during baseline, early ischemia (one minute after balloon inflation in the LAD), early reperfusion (one minute following balloon deflation in the LAD) and late reperfusion (ten minutes following balloon deflation in the LAD).

### Protocol

At baseline, measurements of blood pressure, pulse and APV were performed. Blood pressure and pulse were measured continuously with coronary blood flow and APV analyzed once every ten seconds. A blood gas analysis was performed at baseline and at one and five min post reperfusion

### Reagents

Unless otherwise stated, drugs were purchased from Sigma Co, USA.

### Ethics

The Ethics Committee of Lund University approved the project (M 137-04).

### Calculation and statistics

Calculations and statistics were performed using the GraphPad Prism, version 4.0 software. Values are presented as mean ± s.e.m. Statistical significance was accepted when *P *< 0.05 (two-tailed test). Two-way analysis of variance (ANOVA) test followed by Bonferroni post test was used.

## Results

**Figure 1:**
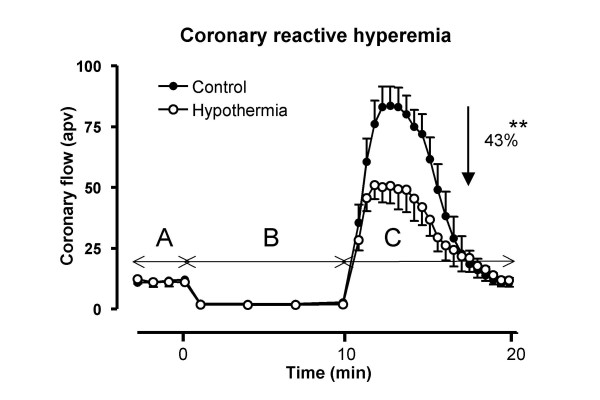
**Coronary reactive hyperemia**. The figure illustrates coronary blood flow expressed as average peak velocity (APV), in cm/s, in the LAD at baseline samples (A), at samples during the LAD occlusion (ischemia) (B), and at 10 sec intervals during the time of the reperfusion period (C), i.e., the phase of post-ischemic reactive hyperemia. The hypothermic group (n = 8) had a significant 43% lower peak flow than the normothermic group (n = 8) during the period of reactive hyperemia. There was no observed difference in coronary flow between the groups during baseline or after 7 minutes from reperfusion.

Coronary blood flow in the LAD increased dramatically during the early reperfusion phase. Peak flow was observed in both groups within 3 minutes following start of reperfusion (deflation of the balloon). The peak flow observed during post ischemic reactive hyperemia was significantly reduced by 43% in the 8 pigs randomized to hypothermia compared to the 8 pigs in the control group (p < 0.01, Figure 1). There was no observed difference in coronary flow between the groups during baseline or after 7 minutes from reperfusion.

**Figure 2:**
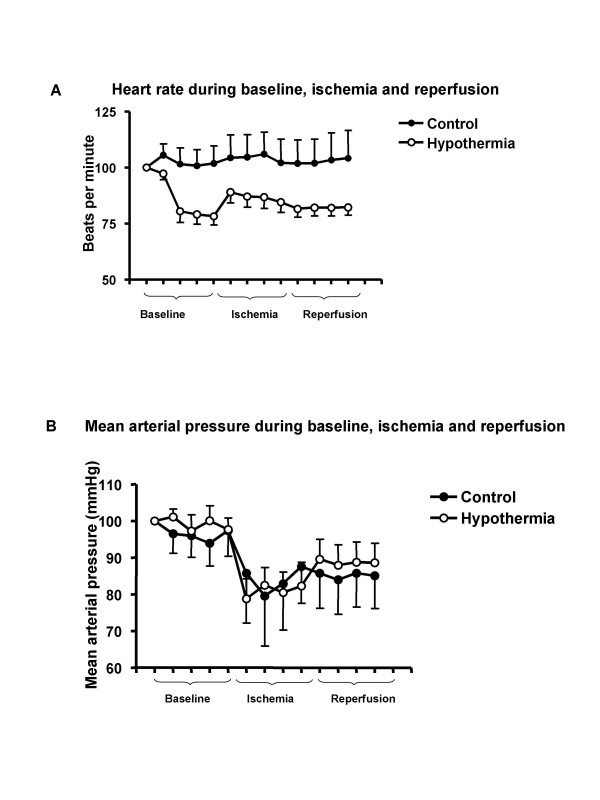
**Heart rate and mean arterial pressure during baseline, ischemia and reperfusion**. Heart rate (HR) was measured during baseline, ischemia, and reperfusion in both the normothermic (n = 8) and the hypothermic (n = 8) pigs. During the entire period of hypothermia HR was lower in the hypothermic group (A). Mean arterial pressure, expressed as mm/Hg, was similar, and reduced in both groups during ischemia and reperfusion compared to baseline (B).

As expected, there was a reduction in heart rate observed among the pigs randomized to the hypothermia group during the entire period of hypothermia (Figure 2A). The difference in heart rate was maintained at the same level during baseline, ischemia and reperfusion, and unaffected by the increased coronary flow measured during reactive hyperemia. Systolic blood pressure was non-significantly reduced in the hypothermia group, but the mean arterial blood pressure (MAP) was similar or even slightly increased in the reperfusion phase compared to the control group (Figure 2b).

**Figure 3:**
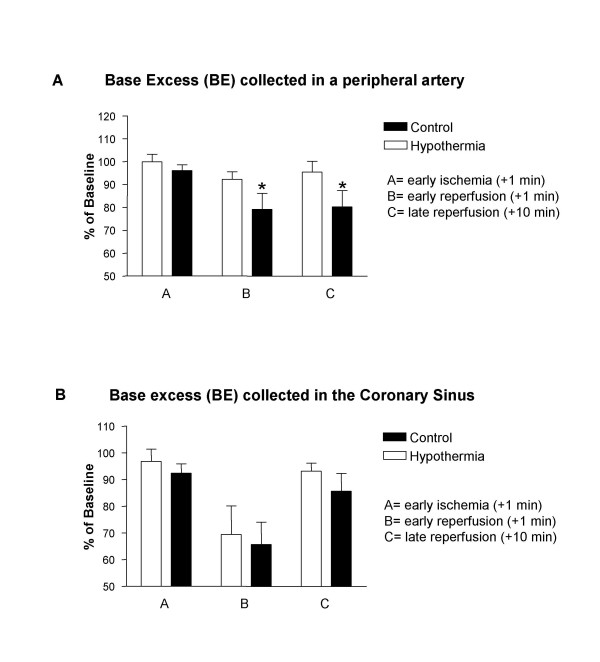
**Base excess (BE) collected in a peripheral artery and the Coronary Sinus**. Base excess (BE) was measured in a peripheral artery (A) and in the Coronary Sinus (CS), (B). There was no difference between normothermic (n = 8) and hypothermic pigs at baseline. During early (+1 minute) and late (+10 minutes) reperfusion there was a significant reduction in BE in the normothermic group in samples from the peripheral artery (A). In samples from the CS, there was a marked reduction in BE in both groups during early reperfusion, which then nearly returned back to baseline at late reperfusion with no difference between the groups (B).

Blood gas analysis showed a significant decrease in peripheral arterial base excess (BE) at one and ten minutes following reperfusion in the control group compared to the hypothermic group, (Figure 3a). Samples collected in the Coronary Sinus showed a prominent reduction in BE in both groups at one minute after reperfusion. BE did not differ significantly between the groups (Figure 3b). Other measurements of blood gas analysis did not show any significant differences in regard to O2 saturation or pH between the groups (not shown).

In separate pigs (n = 4) the diameter of the coronary vessels was measured at the baseline temperature of 37°C (n = 4) and at the hypothermic target temperature of 34°C (n = 2). The vessel diameter was again measured during contrast injection in the LAD in both hypothermic and control pigs during early and late reperfusion. The diameter of the LAD never varied more than 10% from baseline, for each pig, and in our experiments compensation for diameter changes did not markedly influence the results.

## Discussion

We have shown that coronary blood flow during post ischemic reactive hyperemia is reduced nearly by half in the animals treated with mild hypothermia (34°C) compared to controls (37°C).

In general, hypothermia is thought to reduce the metabolic needs of cells and specifically perhaps by reducing the oxygen demand in the hypothermic tissues [21-24]. However, little is known about the actual mechanisms by which hypothermia can reduce cell death in ischemic tissues and the reduced oxygen demand does not fully explain the positive effects of hypothermia [25].

Hypothermia has been shown to limit infarct size if instituted during ischemia and reperfusion [17-19], we therefore postulated that the protective effect of hypothermia is in part mediated by a reduced reperfusion injury. It is still controversial weather reperfusion injury exists as an entity, but also the focus of a great deal of research [1,2]. If there is such a thing as reperfusion injury, one of several events taking place during reperfusion which could potentially cause reperfusion injury is the large increase in coronary flow that occurs during reperfusion (postischemic reactive hyperemia). We therefore wanted to examine if this phenomenon could be affected by hypothermia and thus explain one possible mechanism, or effect, by which hypothermia may exert its protective effect during reperfusion.

The closed chest pig model was chosen to lessen the trauma to the animals and may confer fewer confounding factors than an open chest model. Since the nature of our experiment focused solely on the phenomenon of postischemic reactive hyperemia, we decided on a 10 minute occlusion time which is an established time period for studying reactive hyperemia in both animals and man.

Hypothermia has long been established as a method to limit cell-death due to prolonged ischemia, chiefly in cardiovascular surgery and by neurosurgeons for head and spinal cord injuries as well as during aneurysm repair surgery [26,27]. Recent developments have focused on treating successfully resuscitated, but still comatose, cardiac arrest victims, with hypothermia to improve neurologic outcome. This has led to a revision of guidelines to incorporate cooling of cardiac arrest victims at a class IIb level [28].

Several animal studies have shown that hypothermia reduces infarct size in the setting of myocardial infarct (usually caused by the ligation of LAD). Duncker et al. found a correlation between infarct size and temperature with the smallest infarct size found at the lowest temperature [18]. Other reports have indicated a positive temperature-related effect of hypothermia in relation to infarct size [17,19,20,29-31]. On the contrary Maeng et al. found no benefit of hypothermia induced in conjunction with, or after reperfusion [19]. Others have also shown in small animal models that the earlier hypothermia is applied to ischemic myocardial tissues the lesser the infarct size will be, and that this effect can be further enhanced by preconditioning [20,32]. To date, hypothermia has been tested in five human trials in the setting of myocardial infarction with planned PCI as reperfusion method (COOL MI, ICE-IT, NICAMI [33], LOWTEMP [34] and a study by Dixon et al [35]). The NICAMI and the LOWTEMP studies as well as the study by Dixon were only feasibility trials, whereas the randomized trials COOL MI and ICE-IT were designed to test if a reduction of final infarct at size, by 30-day SPECT analysis, could be attained by treatment with mild hypothermia.

Unfortunately, both COOL MI and ICE-IT were negative trials. At first glance this should have put an end to further cooling trials in the setting of acute MI. However, in both trials, only a minority of patients randomized to cooling reached the target temperature prior to reperfusion, and the few patients that did reach target temperature prior to reperfusion exhibited a significant or strong reduction in myocardial infarct size at 30 days compared to control-patients, especially among patients with anterior infarcts. The result of the two trials is thus in accordance with the results found in preceding animal studies which exemplifies the importance of reaching target temperature prior to reperfusion of completely occluded vessels. A sixth human Trial, COOL MI II [36], has recently been started in lieu of these findings. In this trial, only patients with anterior acute MI's will be randomized to intravascular cooling or control, and cooling will be initiated earlier than in COOL-MI I.

The previous animal and human trials have all looked at the cause and effect of using hypothermia as a means to reduce myocardial infarct size. However, the mechanism by which hypothermia exerts its myocardial protective effect has been explained mainly by a reduction in oxygen consumption [22,23,33,34,37].

Postconditioning as a means to reduce infarct size has recently been successfully performed in several animal experiments and one trial in man [38-41]. There have also been some negative or ambiguous reports concerning the effects of postconditioning [42,43]. Interestingly, the reduced flow often employed during the first couple of minutes (cycles of occlusion and reperfusion) during reperfusion in postconditioning experiments may cause a similar effect to that of the blunted reperfusion flow observed in the very early phase of hypothermia in our experiment. We found that hypothermia reduces the un-proportionally large coronary flow seen during post ischemic reactive hyperemia in the LAD, in a closed chest porcine myocardial infarct model. This finding may explain one of the effects by which hypothermia can reduce infarct size if attained prior to reperfusion.

The reason why hypothermia reduces reactive hyperemia is not fully elucidated. It is probably mediated by a reduction in the release of dilatory mediators, but it could be caused by a reduced responsiveness in the endothelium and vascular smooth muscle cells.

Hypothermia also seems to better maintain BE as measured in the peripheral artery (internal Iliac Artery or distal abdominal Aorta) with no significant differences seen in the coronary venous outflow in the coronary sinus. One could speculate if the difference in BE could have influenced the results of coronary flow during reactive hyperemia. However it is well established that the vascular bed of the coronary circulation tends to dilate in response acidosis and it has been suggested that a decrease in extracellular pH could be involved in coronary flow regulation during hypoxia, ischemia, and increased metabolic demand [44,45]. Thus the significantly lower arterial BE observed in the control group could potentially translate into a higher coronary flow compared to the hypothermic group. However, there was no observed difference in pH, thus making it unlikely that the difference in BE levels would significantly contribute to a substantial difference in coronary flow.

The heart rate (HR) of the pigs in the hypothermic group was clearly lower compared to pigs in the normothermic group during the entire phase of hypothermia. During mild hypothermia in pigs (and humans) a temperature dependent decline in heart rate is accompanied by an increase in stroke volume and may actually increase cardiac output according to Weisser et al. and others [17,46], while on the other hand coronary flow remains unchanged during hypothermia [24]. However, although heart rate declines during hypothermia, this is an independent mechanism unrelated to the cardio-protective effects (i.e. infarct size reduction) of hypothermia [47,48]. In regard to blood pressure, the hypothermic group of pigs showed no difference in mean arterial pressure (MAP) even though a general decline in MAP was observed in both groups mainly during ischemia.

## Conclusion

Hypothermia reduces the un-proportionally large coronary flow seen during post ischemic reactive hyperemia in the LAD, in a closed chest porcine myocardial infarct model. This finding may explain one of the effects by which hypothermia can reduce infarct size if attained prior to reperfusion. Further research into the infarct-reducing mechanisms of hypothermia is warranted if we are to use hypothermia in the clinical setting of myocardial infarction.

## List of abbreviations used

APV Average Peak Velocity

BE Base Excess

CS Coronary Sinus

HR Heart Rate

LAD Left Anterior Descending Artery

## Competing interests

The author(s) declare that they have no competing interests.

## Authors' contributions

GKO conceived, carried out and coordinated the animal study, analysed data, drafted and wrote the manuscript. MG participated in animal experiments and carried out lab analysis and also gave valuable analysis of the manuscript. JH developed the animal model and gave invaluable advice on both the animal experiment and the manuscript. JVDP participated in the animal experiments and gave valuable analysis of the manuscript. DE conceived the study, performed the statistical analysis and wrote the manuscript. All authors read and approved the final manuscript.

## Pre-publication history

The pre-publication history for this paper can be accessed here:
